# Blood-stage *Plasmodium vivax* antibody dynamics in a low transmission setting: A nine year follow-up study in the Amazon region

**DOI:** 10.1371/journal.pone.0207244

**Published:** 2018-11-12

**Authors:** Camilla V. Pires, Jessica R. S. Alves, Barbara A. S. Lima, Ruth B. Paula, Helena L. Costa, Leticia M. Torres, Taís N. Sousa, Irene S. Soares, Bruno A. M. Sanchez, Cor J. F. Fontes, Francis B. Ntumngia, John H. Adams, Flora S. Kano, Luzia H. Carvalho

**Affiliations:** 1 Instituto René Rachou/FIOCRUZ, Belo Horizonte, Minas Gerais, Brazil; 2 Departamento de Análises Clínicas e Toxicológicas, Faculdade de Ciências Farmacêuticas, Universidade de São Paulo, São Paulo, SP, Brazil; 3 Instituto de Ciências da Saúde, Universidade Federal de Mato Grosso, Campus Sinop, Sinop, Mato Grosso, Brazil; 4 Hospital Júlio Muller, Universidade Federal de Mato Grosso, Cuiabá, Mato Grosso, Brazil; 5 Center for Global Health and Infectious Diseases Research, Department of Global Health, College of Public Health, University of South Florida, Tampa, Florida, United States of America; University of São Paulo, BRAZIL

## Abstract

*Plasmodium vivax* remains a global health problem and its ability to cause relapses and subpatent infections challenge control and elimination strategies. Even in low malaria transmission settings, such as the Amazon basin, where progress in malaria control has caused a remarkable reduction in case incidence, a recent increase in *P*. *vivax* transmission demonstrates the continued vulnerability of *P*.*vivax*-exposed populations. As part of a search for complementary approaches to *P*.*vivax* surveillance in areas in which adults are the majority of the exposed-population, here we evaluated the potential of serological markers covering a wide range of immunogenicity to estimate malaria transmission trends. For this, antibodies against leading *P*. *vivax* blood-stage vaccine candidates were assessed during a 9 year follow-up study among adults exposed to unstable malaria transmission in the Amazon rainforest. Circulating antibody levels against immunogenic *P*. *vivax* proteins, such as the Apical Membrane Antigen-1, were a sensitive measure of recent *P*. *vivax* exposure, while antibodies against less immunogenic proteins were indicative of naturally-acquired immunity, including the novel engineered Duffy binding protein II immunogen (DEKnull-2). Our results suggest that the robustness of serology to estimate trends in *P*.*vivax* malaria transmission will depend on the immunological background of the study population, and that for adult populations exposed to unstable *P*.*vivax* malaria transmission, the local heterogeneity of antibody responses should be considered when considering use of serological surveillance.

## Introduction

Malaria remains a public health problem and its global distribution depends on environmental, economic, social and political factors affecting low-income tropical countries [1]. In the Americas, several countries have achieved remarkable reductions in malaria incidence during the 2000–2014 period (Brazil over 75% decrease, Colombia 71.8%), with some of them entering pre-elimination and elimination phases [2]. Despite this, substantial increases in malaria incidence occurred between 2014 and 2016, with *Plasmodium vivax* representing 64% of all malaria cases [3]. *Plasmodium vivax* challenges control strategies because of its ability to cause relapses [4], early production of mosquito-infective stages [5], and significant proportion of subpatent infections, especially in low-transmission settings, such as the Amazon basin [6,7].

In the Amazon rainforest, high heterogeneity in risk factors for *P*. *vivax*, spatial clustering of transmission and subclinical reservoirs, strongly suggest that public health authorities should reformulate malaria control strategies to include a more sensitive parasite detection method for active surveillance [8,9]. Although PCR-based protocols are expected to accurately identify submicroscopic parasite carriers, infected individuals can harbor very low levels of parasitemia, preventing consistent and reliable detection of parasite DNA [9,10]. As more sensitive diagnostic methods that could be deployed in the field are not available, the use of serological surveys to guide control programs has been suggested, as this strategy may provide more reliable estimates of infection exposure and transmission intensity [11–13]. Accordingly, different surveillance approaches, such as serological surveys, need to be evaluated and their effectiveness in different epidemiological settings determined [14–16].

In order to determine whether serology is useful for monitoring temporal changes in low transmission malaria setting, we evaluated, over a 9 year study period, the antibody response of adult individuals from a well-characterized rural Amazon population [17–19] against three leading *P*. *vivax* merozoite-stage vaccine candidate antigens: the Apical Membrane Antigen-1 (AMA-1), that is expressed by merozoites and sporozoites, as a type I integral membrane protein [20]; the 19-kDa C-terminal region of the Merozoite Surface Protein-1 (MSP-1_19_), which is a processed fragment of the MSP-1 polypeptide that remains on the merozoite surface and is carried into the parasitized erythrocyte [21]; and the Duffy Binding Protein region II (DBPII), a key ligand involved in the main *P*. *vivax* reticulocyte invasion pathway [22]. Since DBPII induces both strain-specific and strain-transcending antibody responses [23,24], we included in this study recombinant proteins corresponding to common variants circulating in the Amazon area (DBPII-Sal1 and DBPII-Brz1), as well as a novel engineered DBPII construct (DEKnull-2) associated with broad DBPII antibody responses [25]. During the 9-year period of our cross-sectional surveys, the level of malaria transmission in the study area fluctuated significantly, allowing us to characterize and associate the varying antibody profiles observed with different transmission intensities. In addition, we demonstrate that in the group of subjects with long-term antibody responses, new malaria episodes significantly boosted antibodies to the more immunogenic protein AMA-1. However, in subjects with short-term antibody responses, specific antibodies were not significantly affected by new episodes of blood-stage malaria infection.

## Materials and methods

### Study area and population

**Fig 1:**
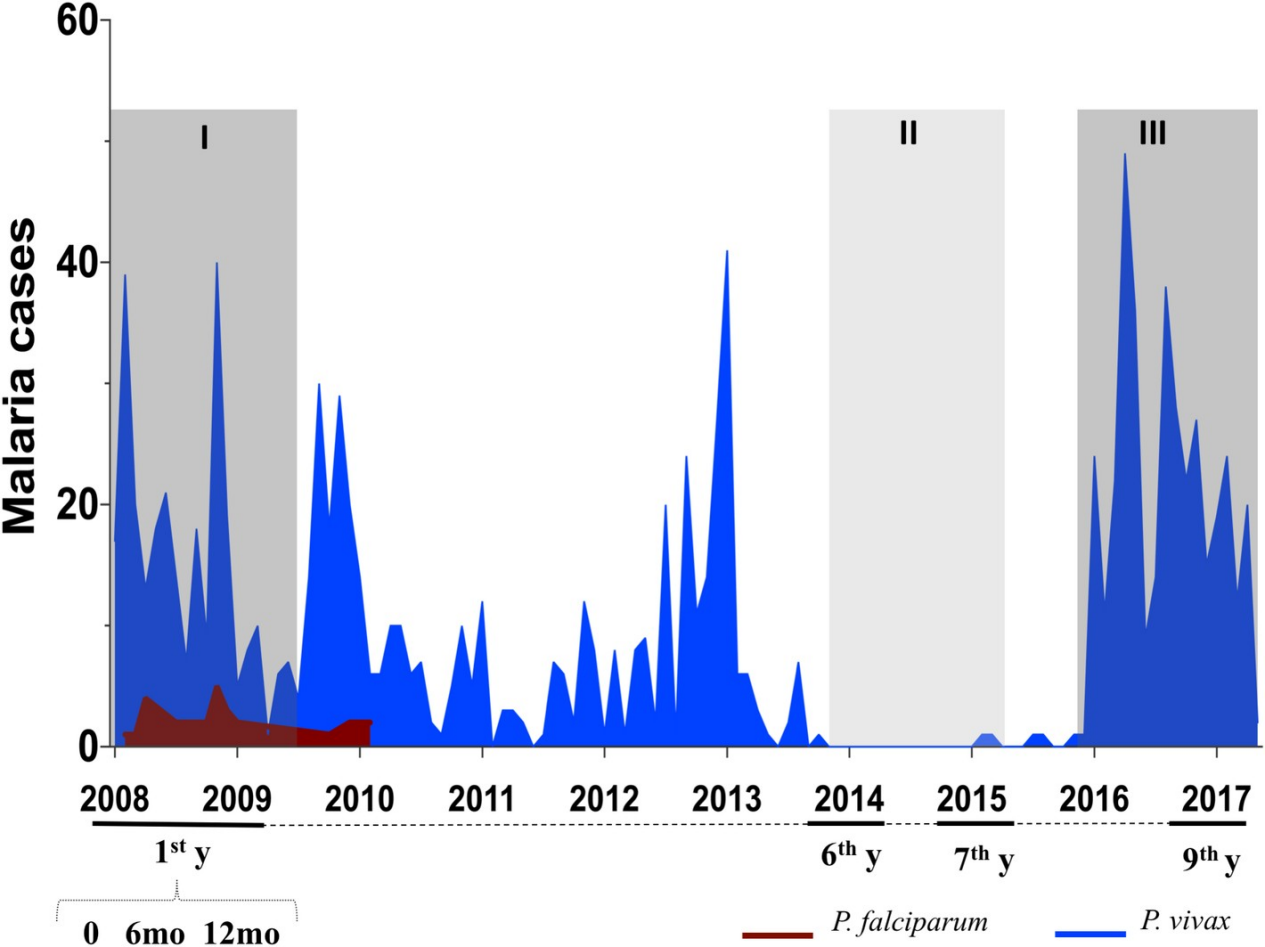
Monthly-time series of malaria cases in the agricultural settlement of Rio Pardo (Amazonas, Brazil) during the study period, 2008–2017. The study design included six cross-sectional surveys distributed in periods of high (dark grey, I and III) and low malaria transmission (light grey, II): three cross-sectional surveys were carried-out during the first year (1^st^ y; enrollment, 6 and 12 months), and three carried-out 6, 7 and 9 years later. Microscopy-diagnosed case report data were provided by the National Malaria Surveillance System Registry (SIVEP-Malaria), with cases of *P*. *falciparum* (red) and *P*. *vivax* (blue) plotted per month.

The study was carried-out in the agricultural settlement of Rio Pardo (1°46’S—1°54’S, 60°22’W—60°10’W), in the municipality of Presidente Figueiredo, state of Amazonas, Brazil. The study site and malaria transmission patterns have been described in detail elsewhere [17–19]. Inhabitants in the settlement live on subsistence farming, and fishing along small streams. In the study area, *P*. *falciparum* malaria incidence has decreased drastically in recent years, and *P*. *vivax* is now responsible for all malaria cases reported (Fig 1).

### Study design and cross-sectional surveys

The ethical and methodological aspects of this study were approved by the Ethical Committee of Research on Human Beings from the Research Institute René Rachou (Reports No. 007/2006, No. 07/2009, No.12/2010, No. 26/2013 and CAAE 50522115.7.0000.5091), according to the Resolutions of the Brazilian Council on Health (CNS-196/96 and CNS-466/2012). In November 2008, 701 residents from the settlement were invited to participate in our study, of which 541 (77.2%) accepted through giving written informed consent, obtained either directly, or from the next of kin, caregivers, or guardians on the behalf of participating minors.

A population-based open cohort study was initiated in November 2008. Six (May-June 2009) and 12 months later (November-December 2009), two similar cross-sectional surveys were undertaken, using the following procedures [17–19]: (i) administration of a structured questionnaire to all volunteers to obtain demographical, epidemiological, and clinical data; (ii) physical examination, including body temperature and spleen/liver size, recorded according to standard clinical protocols; (iii) venous blood collection for individuals aged five years or older (EDTA, 5 mL), or blood spotted onto filter paper (finger-prick) for those aged < 5 years; and (iv) examination of Giemsa-stained thick blood smears for the presence of malaria parasites by light microscopy. The geographical location of each dwelling was recorded using a hand-held 12-channel global positioning system (GPS) (Garmin 12XL, Olathe, KS, USA) with a positional accuracy of within 15 m. Additional cross-sectional surveys were subsequently carried-out 6 (August 2014), 7 (July 2015) and 9 years later (July 2017). Only adults living in the study area were considered eligible for the current study because age-specific seroprevalence rates may not reflect exposure to malaria transmission in the Amazon area [26]. The sample size was calculated based on the ability of the least immunogenic protein to induce antibody responses in 40% of the study population [19]; a minimum sample size of 102 out of 300 adults was required to give a statistical power of 80% (assuming 15% error, a significance level of 5%, and 20% of expected loss).

In the study area, the number of malaria cases fluctuated during the 9-year follow-up period, reflecting periods of high (I and III) and low (II) malaria transmission (Fig 1). The number of microscopically-positive malaria cases were provided by the National Malaria Surveillance System Registry (SIVEP-Malaria). During the study, all microscopically-positive cases were promptly treated as recommended by the Brazilian Ministry of Health. No case of severe malaria was detected in the study area, and all symptoms were of uncomplicated malaria infection (such as headache, chills and myalgia). Overall, the 102 studied subjects contributed a total of 576 plasma samples, with 57 (56%) of them participating in all the long-term cross-sectional surveys conducted after 6, 7 and 9 years, described above.

### Laboratory diagnosis of malaria

At the time of each cross-sectional survey, malaria infections were diagnosed by optical microscopy of Giemsa-stained thick blood smears. The Giemsa-stained smears were evaluated by experienced microscopists, according to the malaria diagnosis guidelines of the Brazilian Ministry of Health. In addition, a non-quantitative real-time PCR using the species-specific amplification of the *18S rRNA* gene of human malaria parasites, was performed. For this, genomic DNA was extracted from either whole blood samples collected in EDTA, or from dried blood spots on filter paper using either the Puregene blood core kit B (Qiagen, Minneapolis, MN, USA) or the QIAmp DNA mini kit (Qiagen), respectively, according to manufacturers’ instructions. The real-time PCR was performed as previously described [27].

### Recombinant blood-stage *P*. *vivax* proteins

#### DBPII-based antigens

Recombinant DBPII proteins included amino acids 243–573 of either the Sal-1 reference strain (DBPII-Sal-1) [28] or the Brazil-1 variant (DBPII-Brz-1), which is highly prevalent in the Amazon region [29]. These proteins were expressed as 39 kDa 6xHis-tag fusion protein, as previously described [30,31]. The Sal-1 sequence was used as a template to create a novel DBPII immunogen (named DEKnull-2) by substituting major DBPII polymorphic residues [25]. The DEKnull-2 design and recombinant antigen production as a carboxyl-terminal 6xHis-tag were described in detail elsewhere [25].

#### MSP-1_19_ antigen

The 19-kDa C-terminal region of the Merozoite Surface Protein-1 of *P*. *vivax* (MSP-1_19_), which represents amino acids 1616–1704 of the full length MSP-1 polypeptide, has been described elsewhere [32].

#### AMA-1 antigen

The ectodomain of *P*. *vivax* Apical Membrane Antigen-1 (AMA-1, encompassing amino acids 43 to 487, were produced as previously described [12,33]. To enable purification, MSP-1_19_ and AMA-1 constructs were also produced as carboxyl-terminal 6xHis_-_tag fusion proteins in a eukaryotic expression system.

### Detection of *P*. *vivax* IgG antibodies by enzyme-linked immunosorbent assay (ELISA)

Conventional enzyme-linked immunoassays (ELISA) for total IgG antibodies to *P*. *vivax* recombinant proteins were carried out as previously described [26], using serum samples at a dilution of 1:100. Recombinant proteins were used at a final concentration of either 3 μg/ml (DBPII Sal-1, DBPII Brz-1 and DEKnull-2) or 1 μg/ml (MSP-1_19_ and AMA-1). For each protein, the results were expressed as ELISA reactivity index (RI), calculated as the ratio of the mean optical density (OD at 492 nm) of each sample to the mean OD plus three standard deviations of samples from 30 unexposed volunteers. Values of RI > 1.0 were considered seropositive.

### Statistical analysis

A database was created using Epidata software (http://www.epidata.dk). The graphics and the analysis were performed using GraphPad Prism version 7 (GraphPad Software, La Jolla California USA, www.graphpad.com) and the R statistical software (version 3.3.2). In the statistical analyses, the antibody response was defined either as a binary categorical variable (the proportion of seropositive individuals) or as a continuous variable (the relative level of individual antibody responses, i.e. the Reactivity Index, RI). Both type of analyses were necessary since antibody levels do not saturate, while seroprevalence does, such that, if transmission intensity declines, antibody levels will drop while seropositivity may remain high [13]. Differences in proportions were evaluated using the chi-square (χ^2^) test or Fisher’s exact tests, as appropriate. The Shapiro-Wilk test was performed to evaluate the normality of the variables. Differences in means and medians were tested, respectively, using either one-way ANOVA, with turkey’s post hoc test, or the Mann-Whitney *U* test or Kruskal-Wallis test, with Dunn’s post hoc test, as appropriate. The linear correlation between variables, such as levels of antibodies and recent episodes of malaria, were determined using the Pearson’s correlation coefficient. For statistical purposes, a recent episode of malaria was defined as a confirmed malaria infection occurring within the 6 month period prior to each cross-sectional survey; for that, all confirmed *P*.*vivax* cases were assessed from the Brazilian Malaria Surveillance System Registry (SIVEP-Malaria, Ministry of Health). *k*-means clustering was used to group the intensity of antibody responses to either the DBP-related antigens (Sal-1, Brz-1 and DEKnull-2), or MSP-1_19_ and AMA-1 combined. A logistic regression model using maximum likelihood was built to describe the independent associations between the intensity of antibody response to each group of antigens (high, moderate and low, as identified by *k*-means clustering) and malaria-exposure variables, such as age, time of residence in the Amazon area, dwelling location (riverine or not), and whether recent vivax-malaria episodes had been reported in the 6 months prior to each cross-sectional survey. The dependent variables of the models were treated as binary values, where the high and moderate response groups was defined as “1”, and the low response group as 0. Two separate logistic regression models were built: one using the *k*-means cluster groups identified using the antibody responses to DBPII, and other using the groups based on MSP-1_19_ and AMA-1. Only variables associated with statistical significance at the 5% level were kept in the final models. As described in the *Results*, we also used a second higher-level classification to categorize responders according to their combined response to both groups of antigens (i.e., DBPII-variants and MSP-1_19_/AMA-1).

## Results

### Antibody profiles to *P*. *vivax* blood-stage antigens at enrollment

Antibodies to the *P*. *vivax* blood-stage antigens were investigated in 102 adults, with a 1:1 male:female ratio, and a median age of 41 years, which correlated with their time of malaria exposure in the Amazon area (median 35 years, r = 0.80, *P* < 0.0001). Overall, antibody responses to MSP-1_19_ and AMA-1 were higher than those to DBPII-related antigens (Table 1). While roughly 70% of individuals had antibodies to MSP-1_19_ or AMA-1, only 40 to 50% of responders had detectable antibodies to DBPII-related antigens, including DEKnull-2 (*P* < 0.005, Fisher’s exact test).

**Table 1:** Epidemiological and immunological data of 102 individuals at enrollment.

CHARACTERISTICS	
**Median age, years (IQR)**	42 (28–53)
**Gender, male:female**	1:1
**Years of residence in Amazon (median, IQR)**	35(24–50)
**Antibody response, positive (%)** ^**†**^:	
***Anti-DBP***_***II***_***Sal1***	50(49)
***Anti-DBP***_***II***_***Brz1***	42(42)
***Anti-DEKnull-2***	52(50)
***Anti-AMA1***	70(68)
***Anti-MSP-1***_***19***_	72(71)

IQR = Interquartile range

† Evaluated by conventional ELISA serology, using recombinant proteins. At enrollment, 14% (15 out of 102) individuals did not have detectable antibodies to any antigen tested, while 34% (35 out of 102) individuals had antibodies to all assayed antigens.

### Temporal-variation of *P*. *vivax* blood-stage antibody responses

**Fig 2:**
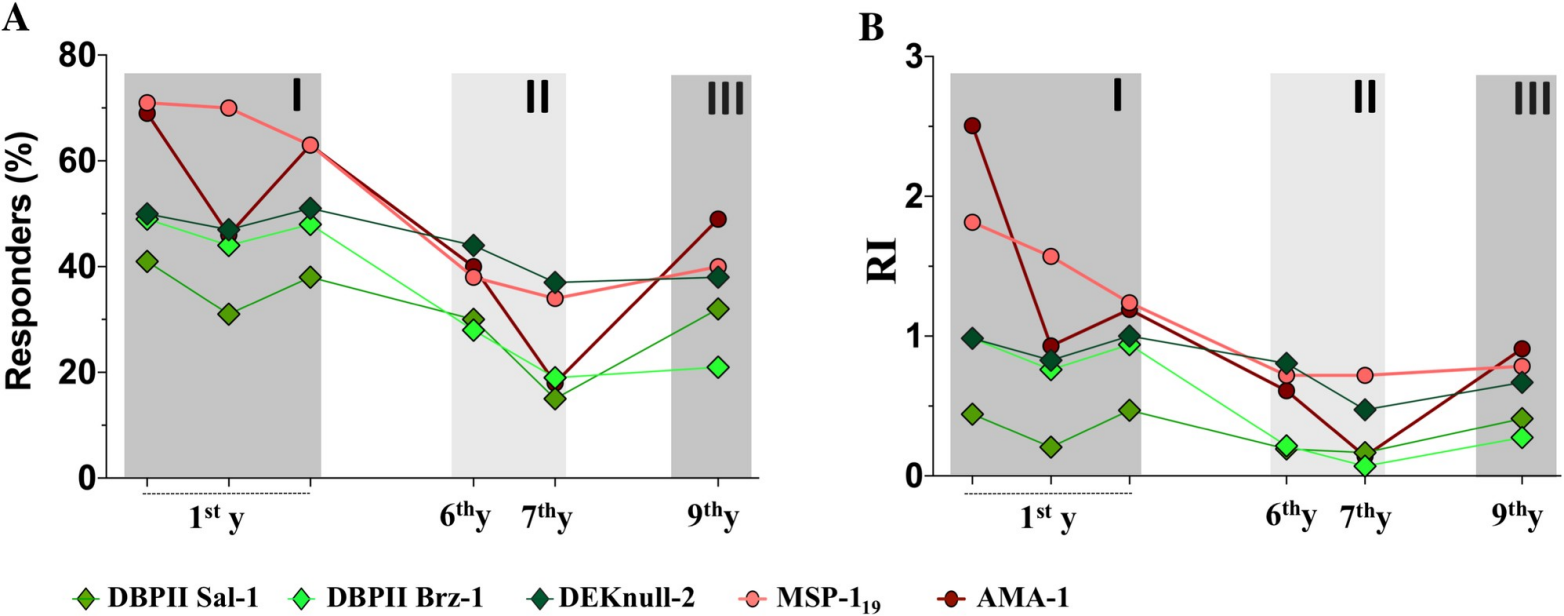
Temporal dynamics of naturally-acquired antibody responses to *P*. *vivax* blood-stage antigens during the study period. The proportion of responders (**A**) and median relative antibody levels (**B**) determined by ELISA using recombinant DBPII-related (DBPII-Sal1, DBPII- Brz1 and DEKnull-2), AMA-1 and MSP-1_19_ antigens. ELISA results were expressed as the medians of the Reactivity index (RI) for each antigen **(B)**, while the proportion of responders **(A)** was determined by considering RI > 1.0 as ELISA-positive response. The cross-sectional surveys were carried-out as described in the legend of Fig 1.

In the study area, the number of malaria cases fluctuated during the 9 year of follow-up, reflecting phases of high (I, III) and low (II) malaria transmission (Fig 1). Accordingly, the proportion of responders and levels of antibodies to *P*. *vivax* blood-stage antigens varied, with greater variation observed among the more reactive proteins, MSP-1_19_ and AMA-1 (Fig 2). In the case of AMA-1, the proportion of responders was significantly associated with the pattern of vivax-malaria transmission, such that the high proportion of responders at enrollment (70%, Phase I) was drastically reduced in the low transmission period (20%, Phase II), but then increased with re-exposure to malaria transmission (50%, Phase III) (*P* < 0.005, Fisher’s exact test) (Fig 2A). The level of anti-AMA-1 antibodies exhibited a similar pattern, with a large decline in the low transmission phase (II) followed by an increase at the end of follow-up period (Fig 2B). Confirming that episodes of *P*. *vivax* malaria infection had boosted AMA-1 antibodies, we found a strong correlation between the levels of anti-AMA-1 antibodies and the number of *P*. *vivax* blood infections occuring during the follow-up period (r = 0.96, *P* = 0.002). Similarly, antibodies to MSP-1_19_ were boosted by *P*. *vivax* episodes (r = 0.86, *P* = 0.02). Despite this, at the end of the follow-up study (Phase III), the average levels of either AMA-1 or MSP-1_19_ antibodies did not reach the baseline levels (Fig 2B).

Although exhibiting much less reactivity than MSP-1_19_/AMA-1, the DBPII-related (Sal-1 and Brz-1) antigens presented a similar profile of antibody responses, including a decrease in both the proportion of responders and relative antibody levels at the time of low malaria transmission (Phase II). On the other hand, the general profile of antibody response to the engineered DEKnull-2 vaccine remained relatively stable throughout the study (Fig 2A), and it was independent of the intensity of local malaria transmission. No significant correlation was found between antibodies to DBPII-related antigens (Sal-1, Brz-1 and DEKnull-2) and the occurrence of *P*. *vivax* blood infections.

### Boosting of antibody levels by blood-stage infection according to previous immunological status

In order to identify variables that could be involved in the presence, intensity and persistence of the antibody responses, we analyzed those 57 individuals who had participated in all phases of the long-term cohort study. The ELISA results were clustered (high, moderate and low by K-means clustering method) according to antigen group, which were grouped as either (i) DBPII-related proteins (Sal-1, Brzl-1, DEKnull-2), or (ii) MSP-1_19_/AMA-1 proteins (S1 Fig). Using logistic regression models (S2 Fig), the total number of *P*. *vivax* malaria episodes reported during the 9 year follow-up period was the only predictor significantly associated with “high/moderate” clusters of response to MSP-1_19_/AMA-1 (OR = 2.05, 95% CI = 1.11–3.78, *P* = 0.02), suggesting that the influence of recent malaria episodes was relevant only in the group of responders who present relatively significant levels of antibody response. The time of malaria exposure, evaluated here by subject’s age and time of residence in the Amazon area, were not significantly associated with the levels of antibody response to any of the panel of antigens used.

**Fig 3:**
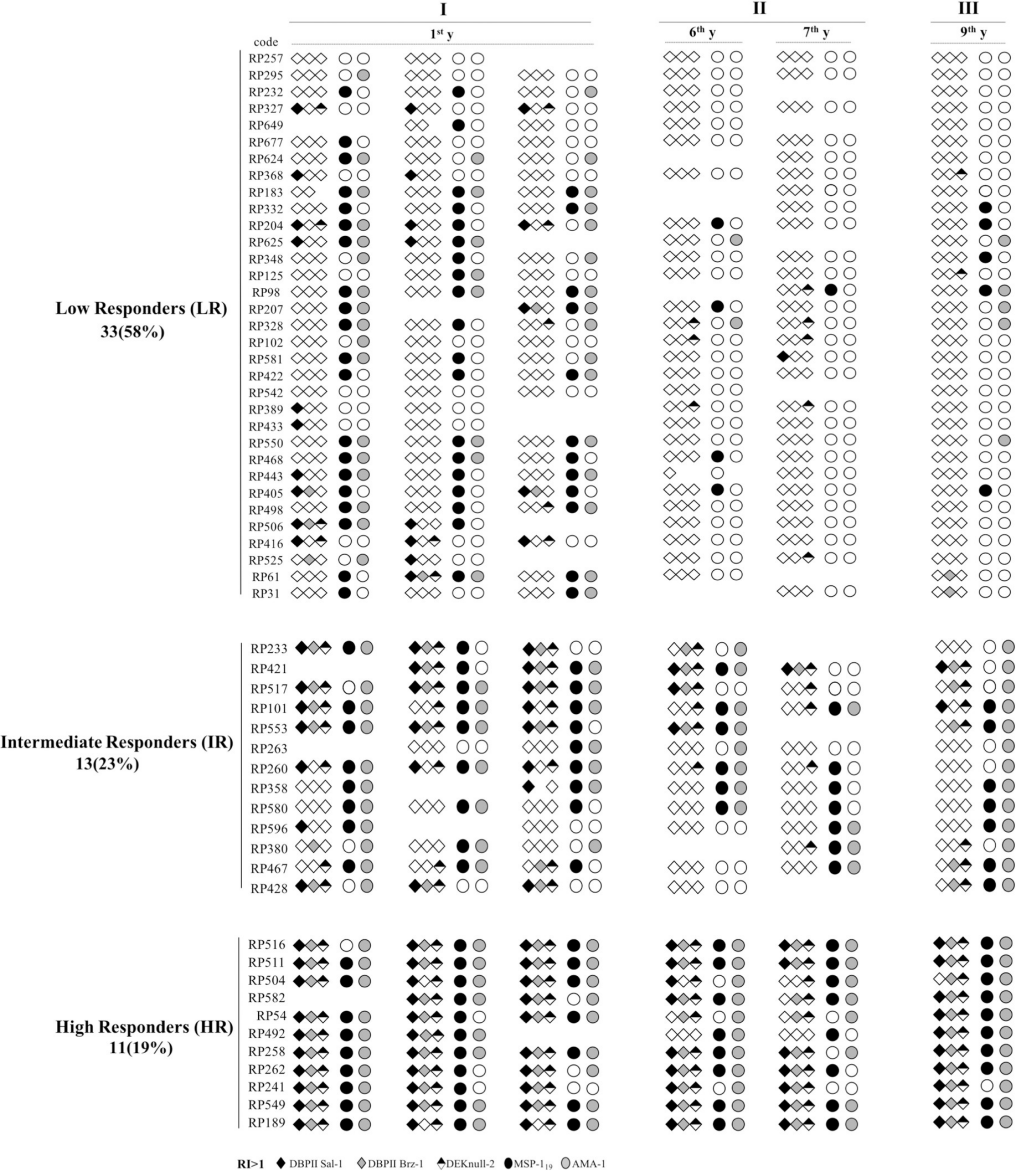
Individual antibody responses to the *P*. *vivax* blood-stage antigens (DBPII-related antigens, MSP-1_19_ and AMA-1) during the cross-sectional surveys. Based on results of the *k*-means clustering (S2 Fig), 57 subjects who had consecutive plasma samples throughout the 9 year follow-up (phases: I, II and III) were further categorized based on their combined responses to both groups of antigens (i.e., DBPII-variants versus MSP-1_19_/AMA-1): (i) *Low responders (LR)*, whose plasma samples had a “low” response to all tested antigens; (ii) *Intermediate responders (IR)*, whose samples gave heterogenous and variable responses to each group of antigens (DBPII–related proteins vs. MSP-1_19_/AMA-1), changing from “low” to “moderate/high” and vice-versa; and (iii) *High responders (HR*), whose samples were clustered as “moderate/high” for both groups of antigens. For each antigen, the antibody-positive response was determined by ELISA (Reactivity index, RI > 1). The blank spaces in the lines correspond to missing samples from the subjects at the time of that cross-sectional survey.

As individuals varied considerably in their antibody responses to the two different groups of antigens (i.e., DBPII-variants versus MSP-1_19_/AMA-1), we also used another higher-level classification to categorize responders according to their combined response to both groups of antigens (Fig 3): (i) *low responders (LR)*, 33 out of the 57 (58%) individuals who present an unstable response of antibodies and whose plasma samples were clustered as “low” to both group of proteins (DBPII-related and MSP-1_19_ & AMA-1); (ii) *intermediate responders (IR)*, 13 (23%) of subjects, exhibiting heterogenous antibody responses, which varied in duration (generally, short-lived to DBPII, and long-lived to MSP-1_19_/AMA1), as well as intensity (being “low” or “moderate/high” to one group of antigens, but not the other); and (iii) *high responders (HR)*, including here 11 (19%) individuals, with persistent long-lived antibody responses, which clustered within the “moderate/high” group for both antigens. Although the HR group was slightly older than the other groups (median 59 vs. 44–53 years), this difference was not statistically significant. However, there was a significant difference between the groups of responders in relation to dwelling location, with the majority of HR members (90%) living close to vector breeding sites (riverine population) as compared with IR or LR groups (38% and 42%, respectively) (Fisher’s exact test, *P* < 0.0001) (Table 2). Through this grouping of the studied subjects, it was possible to show that levels of antibodies to AMA-1, but not MSP-1_19_, were correlated with variation in the intensity of malaria transmission (r = 0.94, *P* = 0.004) (S1D Fig; S3 Fig, IR and HR groups). On the other hand, the response to DBPII-variants was not particularly informative, with the intensity of transmission not significantly associated with a boosting of antibody response to these antigens; of particular importance, the levels of antibodies reacting to DEKnull-2 declined during the study, including in the high transmission phase III. In the LR subgroup, regardless of the antigen assayed, antibody responses were not boosted by episodes of malaria, although significantly fewer were experienced by this group: the percentage of subjects with recent vivax blood-infection in phases I and III for HR (54% and 36%, respectively) were significantly higher than in the LR group (38% and 6%, respectively) (Table 2).

**Table 2:** Epidemiological characteristics of the groups categorized as low responders (LR), IR intermediate responders (IR) and high responders (HR) groups.

	Age, years*	Malaria-exposure, years *^1^	Recent vivax malaria Episodes^2^		Riverine (%)^4^
			Positive diagnostic in “I” (%)^3^	Positive diagnostic in “III” (%)^3^	
**LR**	53(40–62)	46(34–60)	38%	6%	42%
**IR**	44(31–52)	42(31–51)	23%	7%	38%
**HR**	59(40–62)	58(38–62)	54%**	36%***	90%***

*Median (IQR–Interquartile ranger)

** Statistically different (Fisher’s exact test, *P* < 0.05)

***Statistically different (Fisher’s exact test, *P* < 0.0001)

^1^ Time living in the endemic area (Brazilian Amazon)

^2^ Recent malaria was defined by *P*. *vivax* infections recorded up to six months before each cross-sectional survey (2008–2017)

^3^ Recent malaria reported at phases I or III, as defined in legend of Fig 1.

^4^Dwelling located along of local stream (riverine population)

## Discussion

Serology has become an increasingly important tool for the surveillance of a wide range of infectious diseases, including the well-characterized epidemiology of *P*. *falciparum* [13,34]. Using antibody prevalence to *P*. *falciparum* asexual blood-stage antigens, most previous studies estimate seroconversion rates from age-dependent seroprevalence, and have found it a usefull tool for inferring malaria transmission intensity [35,36]. Although this strategy seems to provide reliable estimates of *P*. *falciparum* transmission, equivalent data on *P*. *vivax* malaria are scarce [12,15,37,38]. Even though previous *P*. *vivax* studies have assessed IgG antibodies to a panel of *P*. *vivax* proteins, longitudinal studies are required to better understand the role of serological markers as tools to detect temporal fluctuations in *P*.*vivax* transmission [15]. In the current study, to obtain a reliable estimates of *P*. *vivax* blood-stage antibody dynamics, we assessed antibodies to leading *P*. *vivax* merozoite-stage vaccine candidates–at 6 time-points during a 9 year study period–among malaria-exposed adults living in an area of low but unstable malaria transmission[17–19,39]. We evaluate here the breadth and magnitude of antibody responses independently of subject age because stratification by age seems to be more appropriate for *P*.*falciparum* in Africa [11,13,40]. In *P*. *falciparum-*endemic areas young children bear the major burden of disease [41], while in low transmission settings, such as the Amazon area, age-specific seroprevalence rates may not enable differentiation of recent changes in malaria transmission intensity. Consistent with this, no association was found here between *P*. *vivax* blood-stage antibodies and either the age of studied subjects or their time living in the endemic area.

Although we demonstrated here that AMA-1 and MSP-1_19_ were comparable in immunogenicity, the current study confirmed previous findings showing that AMA-1 seems to be a sensitive marker to detect fluctuations in areas of unstable malaria transmission [12,42–44]. Accordingly, for a wide range of transmission settings AMA-1, but not MSP-1_19,_ provided to be a much more precise estimator of malaria transmission [13]. In *P*.*falciparum* studies, recent findings also confirm that AMA-1 is a useful surrogate marker in low transmission settings [45]. Additionally, our results indicate that the applicability of circulating blood-stage antibodies as serological markers for recent infection depends on the immunological background of the study population. Thus, in a group of age-matched individuals with similar durations of exposure to unstable malaria transmission, we found that the antibody profile of responders varied greatly, enabling the identification of different patterns of antibody response. Interestingly, only individuals who had a robust and long-lived response to MSP-1_19_/AMA-1 cluster (HR and IR groups) exhibited a straightforward relationship between AMA-1 antibody boosting and *P*. *vivax* blood-stage episodes. Also, while HR and IR groups could be differentiated by breadth (specificities for multiple antigens) of antibody responses, the profile of AMA-1 antibodies remained relatively similar between these groups of responders. These findings suggest that in the studied subjects, the levels of AMA-1 antibodies (but not the breadth of antibody responses *per se*) is useful as a serological marker of long-term changes in malaria transmission. On the other hand, it has been suggested that an increasing breadth of antibody specificities could be dependent upon the level of antigenic input [37], with high-levels of antibodies to multiple antigens indicative of clinical protection [46]. Although the protective nature of the antibody responses was not the focus of the current work, it seems reasonable to assume that the level of acquired immunity to *P*. *vivax* malaria is probably much higher in the HR group than in IR group. The majority of HR subjects live close to vector-breeding sites (riverine population), being more exposed to malaria transmission, and heterogeneity in mosquito exposure contributes considerably to heterogeneity in infection risk [47]. In our study area, the probability of being infected is five-times higher in people living along the Rio Pardo stream than in those living along the unpaved forest roads [17]. Consequently, subjects classified as HR may have been exposed sufficiently, within the study area, to induce a stable long-lived IgG response to many proteins, including DBPII-strain specific antigens (Sal-1 and Brz-1), whose response tend to be short-lived and biased towards strain-specific antibodies [24,48]. However, the low number of subjects classified as HR in our study precludes any definitive conclusion about the profile of antibody response and clinical protection. To properly investigate the acquisition of antibody-mediated protection, it is more appropriate to use inhibitory assays to evaluate the presence of *P*. *vivax* neutralizing antibodies [49].

*Plasmodium vivax* antigens spanning a range of immunogenicities were included in the current study in order to build up a detailed picture of the immunological background of the studied individuals. At both individual and population levels, DBPII-strain-specific antigens were not clearly associated with variation in malaria transmission over time. This finding was not completely unexpected since in low transmission settings, such as the current study area, serological markers based on highly immunogenic antigens seem to be more appropriate than less immunogenic antigens [11]. To overcome the inherent DBPII bias towards developing strain-specific immunity, the methodological approach also included a novel DBPII immunogen, DEKnull-2, as it has been shown to elicit a stronger, broader and long-term neutralizing antibody response, due to the elimination of strain-specific polymorphisms associated with poor immunogenicity [25]. However, at the population level, the DEKnull-2 seroprevalence reached a plateau during the first year (40–50% of responders), and the frequency of response remained stable throughout the study. Although the phenomenon of saturation of antibody response has been well documented, it seems to be much more common for highly immunogenic antigens in areas of moderate to high *P*. *falciparum* transmission [11]. By using a DBPII constructs designed to induce broad antibody responses, we observed a similar pattern of antibody saturation, but in an area of relatively low and unstable *P*. *vivax* transmission. In agreement with these findings, it has been suggested that antibody titers to *P*. *vivax* antigens tend to reach their corresponding plateau sooner than its *P*. *falciparum* counterparts [12]. Although it is not clear why antibodies to *P*. *vivax* antigens tend to reach their corresponding plateau sooner, it has been speculated that it may be related to hypnozoite infection [50], and the apparently faster acquisition of clinical immunity to *P*. *vivax* compared to *P*. *falciparum* [51]. Aditionally, we have previously demonstrated that the prevalence and persistence of DBPII antibody response are largely influenced by HLA Class II allelic variants [19]. Thus, while the DEKnull-2 antibody response is not a useful tool for serological evaluation of malaria transmission, it seems to be appropriate to evaluate naturally-acquired immunity to *P*. *vivax* blood-stage infections. At the individual level, DEKnull-2 immune responses confirmed the highest acquired immunity of HR group as compared with IR or LR group; all HR subjects were positive to DEKnull-2, and remained seroreactive until the end of the 9 year follow-up study. Despite of that, the levels of antibodies reacting to DEKnull-2 declined during the study, including in the high transmission phase III.

As the current study involved a relatively long-term follow-up, it was only possible to obtain samples from 57 out of 102 individuals for the full 9 year study period, which resulted in a decrease in the statistical sample power from 80% to 63%. Despite this, the profile of antibody response remained as expected. Even though our limited sample size precluded a more robust statistical analysis, the results presented here confirm that in the study area the levels of AMA-1 IgG antibodies fulfill the prerequisite to be used as a marker of *P*. *vivax* transmission intensity, as levels are boosted by re-exposure to parasite blood-stages, and dropped when transmission is reduced. In an area of unstable malaria transmission, such as the Amazon area, its efficiency will depend on immunological status of the studied individuals, since a certain level of prior exposure appears to be necessary for adequate boosting of the antibody response. Thus, for adult populations exposed to unstable *P*. *vivax* malaria transmission, the heterogeneity of responders needs to be considered when planning whether to use serological markers as a complementary approach to increase the effectiveness of estimating malaria transmission.

## Supporting information

S1 Fig **Clustering of the antibody responses to DBPII variants (A) and MSP-1_19_ and AMA-1 (B)**. The *k*-means clustering method was used the identify clusters according the ELISA reactivity index (RI). For the analysis, the 57 malaria-exposed subjects who participated until the end of the follow-up period (phases, I, II and III), and for who there were multiple consecutive samples, were included. For each group of proteins, three clusters were identified (high, moderate and low). C and D show the RI (median and interquartile ranger, IQR) for DBPII-related antigens and MSP_19_ & AMA-1, respectively, according to the *k*-cluster of reactivity.
(TIF)

S2 Fig **Logistic regression models describing the association between vivax malaria episodes and antibody responses to MSP-1_19_ and AMA-1 (A) but not to DBPII-related antigens (B).** While the predicted probability of the MSP-1_19_ and AMA-1 antibodies levels increases significantly with new episodes of *P. vivax* malaria (*P < 0.01), DBPII-related antigens were not associated. The variables age, time of residence in Amazon Region, and dwelling location were also included in the logistic models but were not significantly associated (data not shown).
(TIF)

S3 Fig Fluctuation in the levels of antibodies to *P. vivax* blood-stage antigens according to groups of individuals defined by their combined responses to both groups of antigens (i.e., DBPII-variants versus MSP-1_19_/AMA-1). Three groups of responders were identified as Low (LR), Intermediate (IR) and High (HR) responders, as described in the legend to Fig 3. The levels of antibody responses were determined by ELISA using recombinant AMA-1 and MSP-1_19_, and DBPII-related antigens (DBPII-Sal1, DBPII- Brz1 and DEKnull-2). For each antigen, ELISA results were expressed as the median of the Reactivity index (RI). The cross-sectional surveys were carried-out as described in legend of Fig 1.
(TIFF)

S1 File Serology database. Code, Reactivity Indexes (ELISA assay) of DBPII Sal1, DBPII Brz1, DEKnull-2, AMA-1 and MSP1_19_, and also recent malaria episodes, for each cross-sectional survey (0, 6 and 12 months, and 6, 7 and 9 years).
(XLSX)
