# Supplementary figures and images for: Blood-stage Plasmodium vivax antibody dynamics in a low transmission setting: A nine year follow-up study in the Amazon region
Source: PLoS One. 2018 Nov 12;13(11):e0207244. doi: 10.1371/journal.pone.0207244 (PMC6231651; doi:10.1371/journal.pone.0207244)

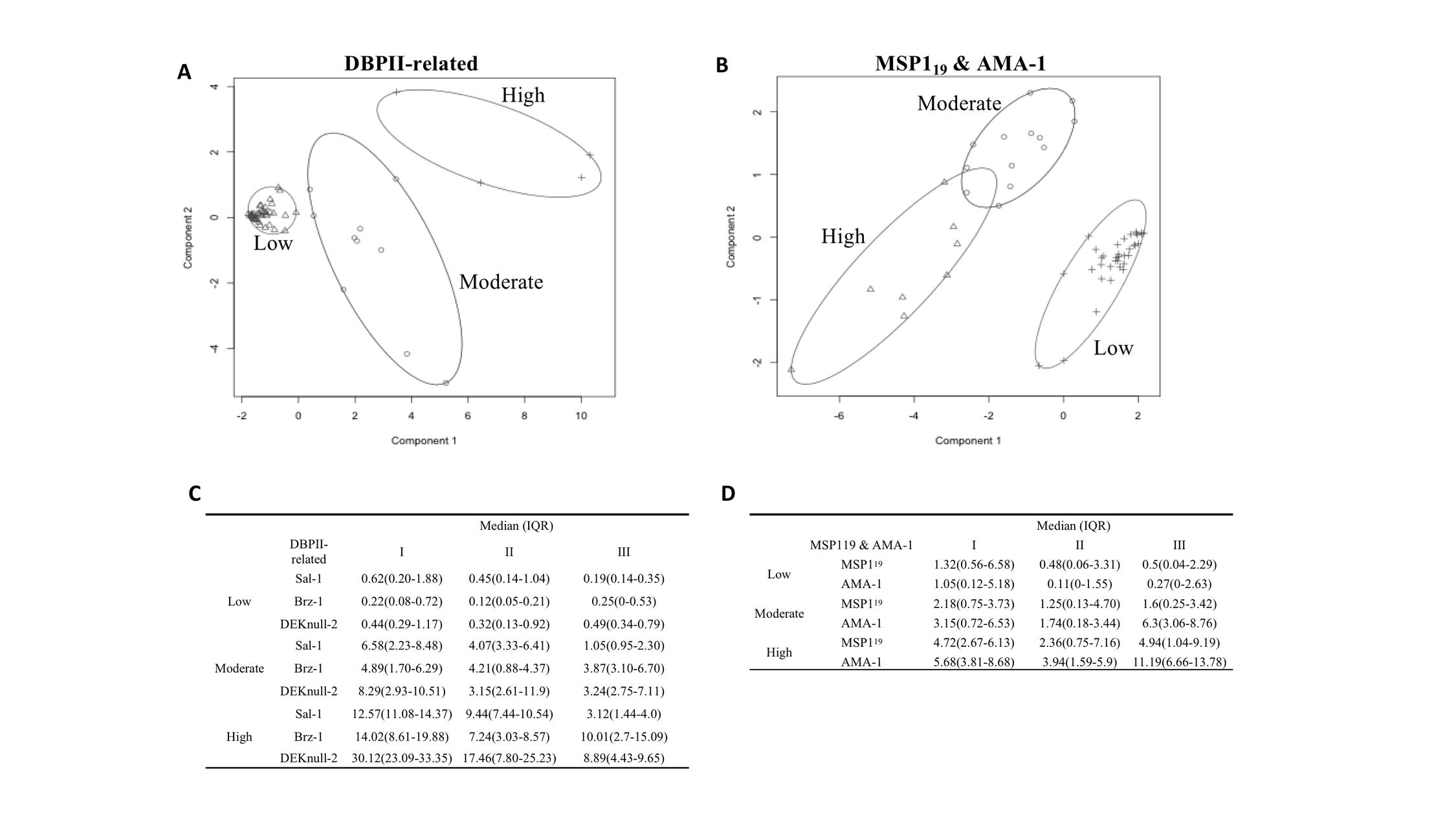

Supplement: S1 Fig — Clustering of the antibody responses to DBPII variants (A) and MSP-119 and AMA-1 (B). The k-means clustering method was used the identify clusters according the ELISA reactivity index (RI). For the analysis, the 57 malaria-exposed subjects who participated until the end of the follow-up period (phases, I, II and III), and for who there were multiple consecutive samples, were included. For each group of proteins, three clusters were identified (high, moderate and low). C and D show the RI (median and interquartile ranger, IQR) for DBPII-related antigens and MSP19 & AMA-1, respectively, according to the k-cluster of reactivity. (TIF) [file pone.0207244.s001.tif]

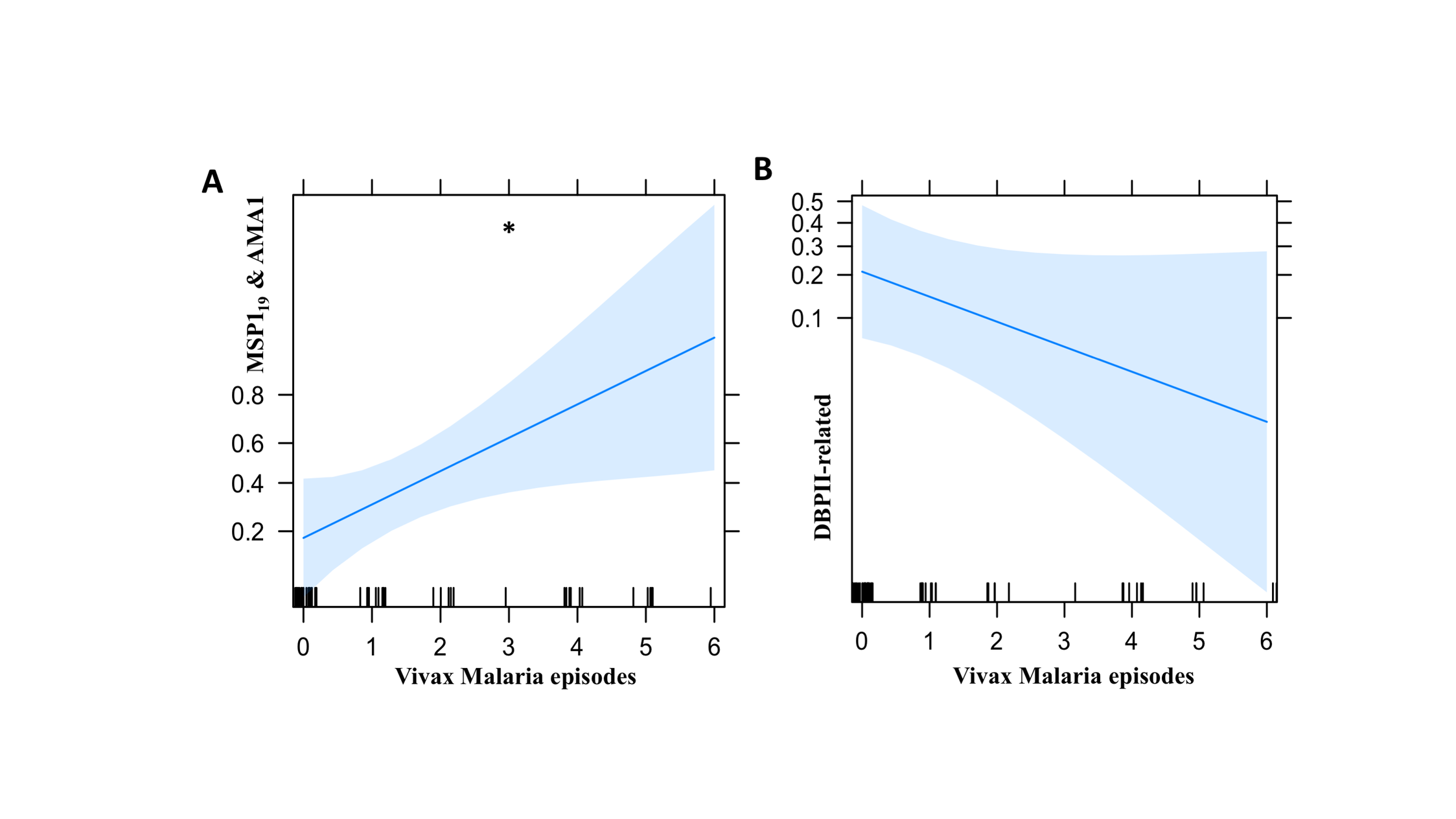

Supplement: S2 Fig — Logistic regression models describing the association between vivax malaria episodes and antibody responses to MSP-119 and AMA-1 (A) but not to DBPII-related antigens (B). While the predicted probability of the MSP-119 and AMA-1 antibodies levels increases significantly with new episodes of P. vivax malaria (*P < 0.01), DBPII-related antigens were not associated. The variables age, time of residence in Amazon Region, and dwelling location were also included in the logistic models but were not significantly associated (data not shown). (TIF) [file pone.0207244.s002.tif]

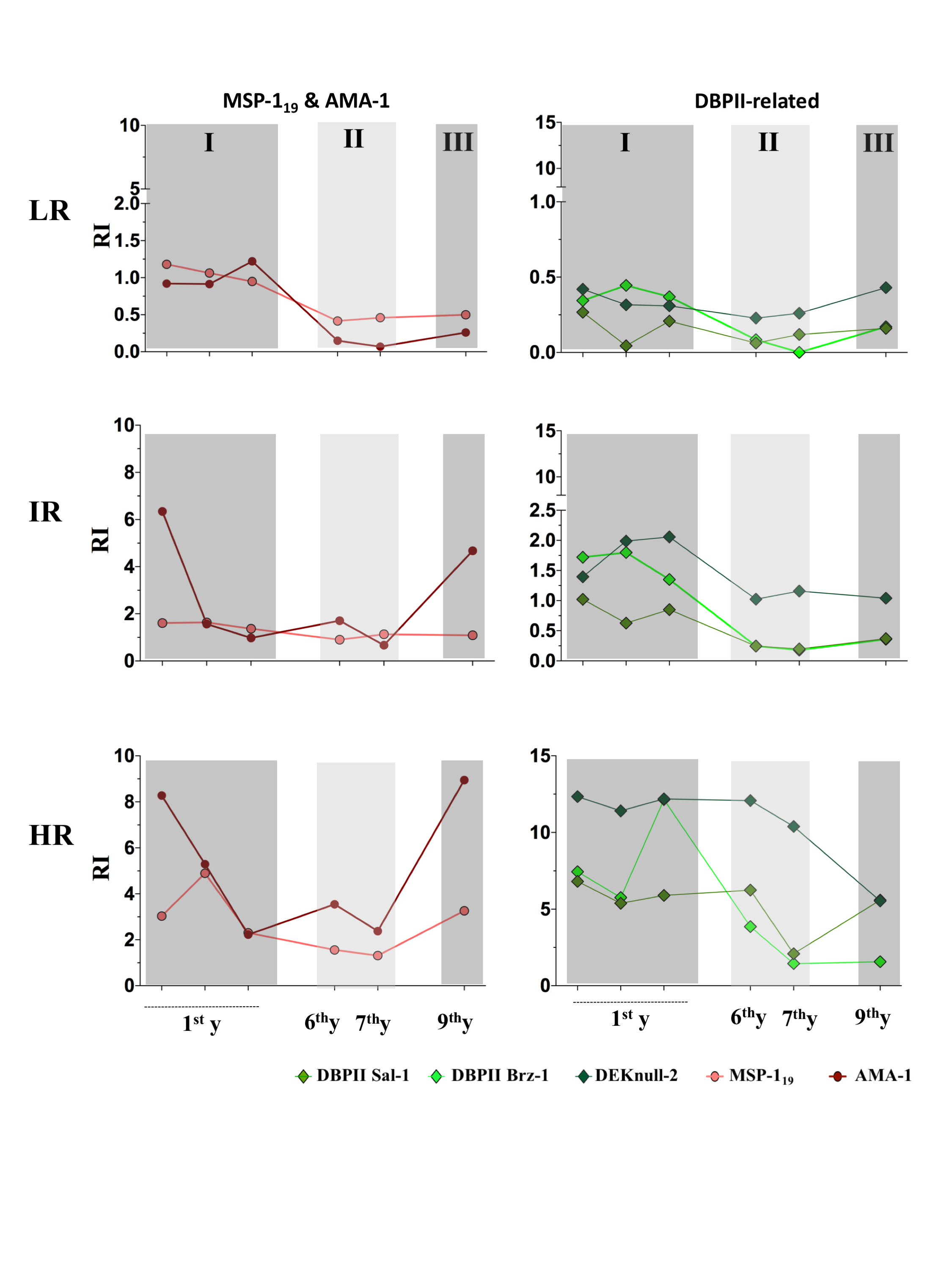

Supplement: S3 Fig — Three groups of responders were identified as Low (LR), Intermediate (IR) and High (HR) responders, as described in the legend to Fig 3. The levels of antibody responses were determined by ELISA using recombinant AMA-1 and MSP-119, and DBPII-related antigens (DBPII-Sal1, DBPII- Brz1 and DEKnull-2). For each antigen, ELISA results were expressed as the median of the Reactivity index (RI). The cross-sectional surveys were carried-out as described in legend of Fig 1. (TIFF) [file pone.0207244.s003.tiff]
